# An Analysis of Plan Robustness for Esophageal Tumors: Comparing Volumetric Modulated Arc Therapy Plans and Spot Scanning Proton Planning

**DOI:** 10.1016/j.ijrobp.2016.01.044

**Published:** 2016-05-01

**Authors:** Samantha Warren, Mike Partridge, Alessandra Bolsi, Anthony J. Lomax, Chris Hurt, Thomas Crosby, Maria A. Hawkins

**Affiliations:** ∗ Cancer Research UK/Medical Research Council Oxford Institute for Radiation Oncology, Gray Laboratories, University of Oxford, Oxford, United Kingdom; † Centre for Proton Therapy, Paul Scherrer Institute, Villigen, Switzerland; ‡ Wales Cancer Trials Unit, School of Medicine, Heath Park, Cardiff, United Kingdom; § Velindre Cancer Centre, Velindre Hospital, Cardiff, United Kingdom

## Abstract

**Purpose:**

Planning studies to compare x-ray and proton techniques and to select the most suitable technique for each patient have been hampered by the nonequivalence of several aspects of treatment planning and delivery. A fair comparison should compare similarly advanced delivery techniques from current clinical practice and also assess the robustness of each technique. The present study therefore compared volumetric modulated arc therapy (VMAT) and single-field optimization (SFO) spot scanning proton therapy plans created using a simultaneous integrated boost (SIB) for dose escalation in midesophageal cancer and analyzed the effect of setup and range uncertainties on these plans.

**Methods and Materials:**

For 21 patients, SIB plans with a physical dose prescription of 2 Gy or 2.5 Gy/fraction in 25 fractions to planning target volume (PTV)_50Gy_ or PTV_62.5Gy_ (primary tumor with 0.5 cm margins) were created and evaluated for robustness to random setup errors and proton range errors. Dose–volume metrics were compared for the optimal and uncertainty plans, with *P*<.05 (Wilcoxon) considered significant.

**Results:**

SFO reduced the mean lung dose by 51.4% (range 35.1%-76.1%) and the mean heart dose by 40.9% (range 15.0%-57.4%) compared with VMAT. Proton plan robustness to a 3.5% range error was acceptable. For all patients, the clinical target volume D_98_ was 95.0% to 100.4% of the prescribed dose and gross tumor volume (GTV) D_98_ was 98.8% to 101%. Setup error robustness was patient anatomy dependent, and the potential minimum dose per fraction was always lower with SFO than with VMAT. The clinical target volume D_98_ was lower by 0.6% to 7.8% of the prescribed dose, and the GTV D_98_ was lower by 0.3% to 2.2% of the prescribed GTV dose.

**Conclusions:**

The SFO plans achieved significant sparing of normal tissue compared with the VMAT plans for midesophageal cancer. The target dose coverage in the SIB proton plans was less robust to random setup errors and might be unacceptable for certain patients. Robust optimization to ensure adequate target coverage of SIB proton plans might be beneficial.

**Summary**

Comparing spot-scanning proton therapy single-field optimization plans with volumetric modulated arc therapy plans indicated that single-field optimization can achieve significant sparing of normal tissue for midesophageal cancer compared with volumetric modulated arc therapy. However, the boost volume dose coverage in the simultaneous integrated boost proton plans appears less robust to setup errors. Robust optimization to ensure adequate target coverage of simultaneous integrated boost proton plans might be beneficial.

## Introduction

Radiation therapy (RT) is an important component in the management of esophageal cancer, for both preoperative and definitive treatment, although the 5-year survival rates in the United Kingdom have been only 12% (1). A meta-analysis of preoperative chemoradiation therapy suggested a radiation dose–response relationship for improved pathologic remission (2) and has provoked interest in RT dose escalation to improve outcomes 3, 4, 5.

Advanced RT techniques such as intensity modulated RT (IMRT) 3, 4 and volumetric modulated arc therapy (VMAT) (6) offer opportunities for increasing the dose to the tumor, although normal tissue sparing for some patients might be limited by the anatomy (5). Proton therapy is thought to improve sparing of normal tissues. Preliminary clinical outcomes with passive scattering proton therapy (PSPT) suggest that 50.4 Gy (relative biological effectiveness (RBE)) can be safely delivered with concurrent chemotherapy 7, 8. Also, the results of a planning study have suggested that using 2 or 3 proton beams with single-field optimization (SFO) spot scanning proton therapy for esophageal cancer could improve lung and heart sparing compared with photon RT (9).

Nonetheless, planning studies to compare x-ray and proton techniques and to select the most suitable technique for each patient have been hampered by the nonequivalence of several aspects of treatment planning and delivery. A fair comparison should compare similarly advanced delivery techniques from current clinical practice and should also assess the robustness of each technique. Therefore, we chose to compare SFO proton plans and VMAT plans 10, 11. SFO plans using pencil beam scanning have been shown to generate a more robust target dose distribution for brain and spine, prostate, and head and neck tumors than multifield-optimized intensity modulated proton therapy plans, when the dose distributions are obtained without robust optimization 12, 13.

The principal factor affecting plan robustness is setup error due to interfraction variations in patient position. Daily cone beam computed tomography (CT) image guidance is routinely used for x-ray RT, and although on-line volumetric image guidance for proton therapy is not yet feasible in clinical practice, daily beam's eye view images would allow patient positioning with bony anatomy or fiducial markers as a reference (14). Additional uncertainty is present for proton beams, arising from the uncertainty in CT number and conversion to proton stopping power, which can modify the proton range in the patient (15).

The present study therefore examined the robustness of the VMAT and SFO dose distributions for a range of patients with midesophageal cancer, assessing the effect of setup error and additional proton range uncertainty on the dose–volume metrics for the target and normal tissues.

## Methods and Materials

**Fig. 1:**
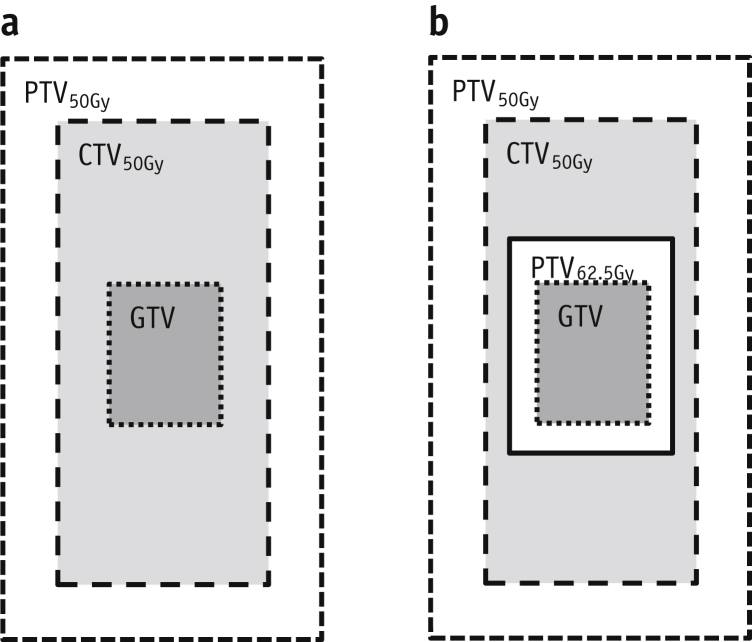
Target volumes and dose prescription for (a) standard and (b) simultaneous integrated boost plans. *Abbreviations:* CTV = clinical target volume; GTV = gross tumor volume; PTV = planning target volume.

**Fig. 2:**
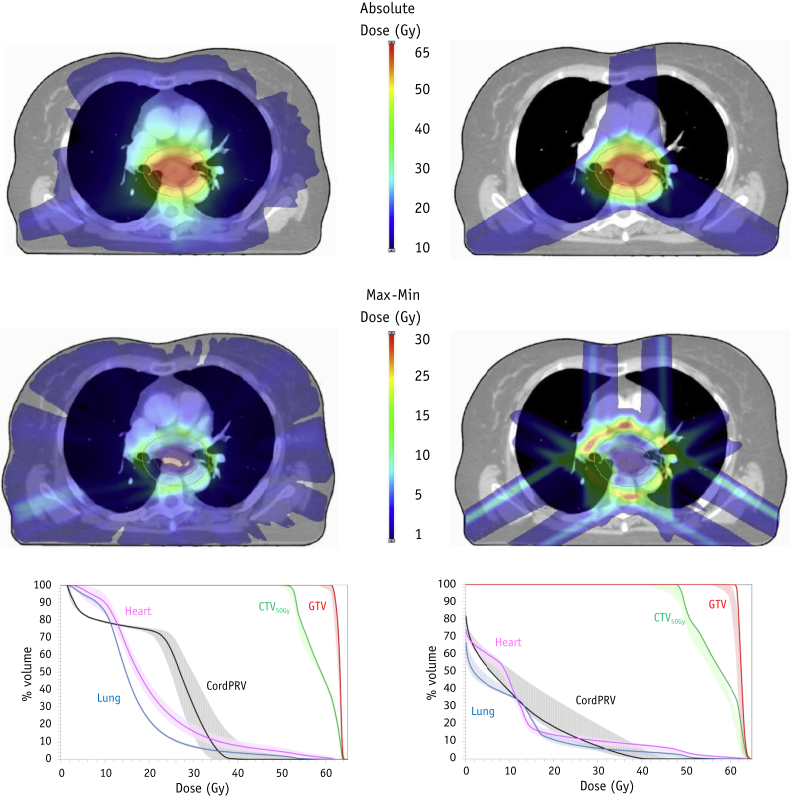
(Top) Example of dose distribution for 1 patient for volumetric arc therapy (VMAT) (Left) and single-field optimization (SFO) (Right) plans. (Middle) The maximum (Max) to minimum (Min) dose difference for the uncertainty plans for VMAT (Left) and SFO (Right). (Bottom) Dose–volume histogram showing the dose to the target volumes and organs-at-risk for VMAT (Left) and SFO (Right). Shaded regions indicate the envelope of maximum and minimum dose from the uncertainty plans.

A subset of 21 patients with midthoracic esophageal cancer was selected from the SCOPE1 clinical trial (ISRCTN 47718479) database (in which the mean planning target volume (PTV) for the entire cohort was 334 cm^3^). The 21 patients selected as representative had a PTV range of 140 to 591 cm^3^ and a mean PTV of 327 cm^3^. The SCOPE protocol standard margins (16) were reapplied to the trial-derived gross tumor volumes (GTVs). The GTV was increased manually 2 cm superoinferiorly along the esophageal axis, with an additional 1 cm radial margin to create the clinical target volume (CTV_50Gy_). An additional 1 cm margin was applied to create the planning target volume (PTV_50Gy_), which was prescribed a dose of 2 Gy/fraction for 25 fractions. The boost volume CTV_62.5Gy_ was considered identical to the GTV, with PTV_62.5Gy_ created by adding an isotropic 0.5 cm margin. This was prescribed a simultaneous integrated boost dose of 2.5 Gy/fraction (Fig. 1). A 0.5 cm margin is the minimum value calculated from random and systematic errors recorded when portal imaging of bony anatomy is used for image guidance in esophageal cancer (17). This margin allows for dose falloff from 62.5 to 50 Gy and minimizes the volume of tissue irradiated to >50 Gy. Visual assessment of normal tissue contours was performed, with data imported into Eclipse, version 13 (Varian, Palo Alto, CA). Two treatment plans were then created for each patient: a RapidArc (VMAT) plan using two 360° arcs of 6 MV and a spot scanning proton therapy plan (SFO) of 70 to 250 MeV using the 3-field beam arrangement described by Welsh et al (9) with single-field optimization (Fig. 2, top row). Both plans were created in physical dose (Gy; ie, proton RBE fixed at a constant value of 1.1), and the beams were optimized using all the constraints listed in Table 1, to achieve the correct target coverage where possible. For proton plans, a proximal and distal range margin (range 0.3-0.5 cm) was added to the PTV_50Gy_. A PTV_62.5Gy_ median dose (D_50%_) was used for plan normalization in the standard plans and showed no statistically significant difference between the VMAT and SFO plans. Plan robustness was evaluated for both VMAT and SFO techniques, using the plan uncertainty tool provided in Eclipse, version 13. This tool simulates setup and range errors and recalculates a new dose distribution for each perturbed plan. Setup errors of *x* (left to right) ±0.5 cm, *y* (craniocaudal) ±0.7 cm, and *z* (anteroposterior) ±0.5 cm were used to generate 6 perturbed plans, representing the maximum expected interfraction setup error for VMAT and SFO. These simulations encompassed the random setup error observed in online image guidance protocols for esophageal cancer (17). For the proton plans, the effect of a range error of ±3.5% was simulated separately (18). Dose–volume metrics for the total dose (62.5 Gy in 25 fractions) for the nominal VMAT and SFO plans were compared across all patients using the Wilcoxon signed rank test in SPSS, version 20.0.0 (IBM Corp, Armonk, NY), with *P*<.05 considered significant. For each patient, the robustness of the VMAT and SFO plans to the setup errors and the SFO plans to range error were compared as the dose per fraction.

## Results

### Nominal plans

The VMAT plans met the dose constraints for 16 of 21 patients. The mean heart dose was exceeded for 3 patients (patients 7, 11, and 17), and the lung V_20Gy_ limit was not met for patients 16 and 21. For patient 21 (with the largest PTV of 590.8 cm^3^), it was challenging to achieve the desired PTV_50Gy_ V_95%_ because of the overlap with lung tissue, although coverage of the CTV_50Gy_ was adequate for this patient, a D_98%_ of 49.1 Gy (Table E1; available online at www.redjournal.org). Except for this patient, the PTV_50Gy_ V_95%_ was 95.8% to 99.9%. For the nominal VMAT plans across all 21 patients, the mean heart dose was 21.2 Gy (median; range 14.4-29.8), mean lung dose was 13.6 Gy (range 8.4-18.1), lung V_20_ was 15.6% (range 5.8%-29.7%), and maximum cord dose (0.1 cm^3^) was 33.3 Gy (range 21.5-36.5). The dose–volume metrics for each patient are provided in Table E1 (available online at www.redjournal.org).

**Fig. 3:**
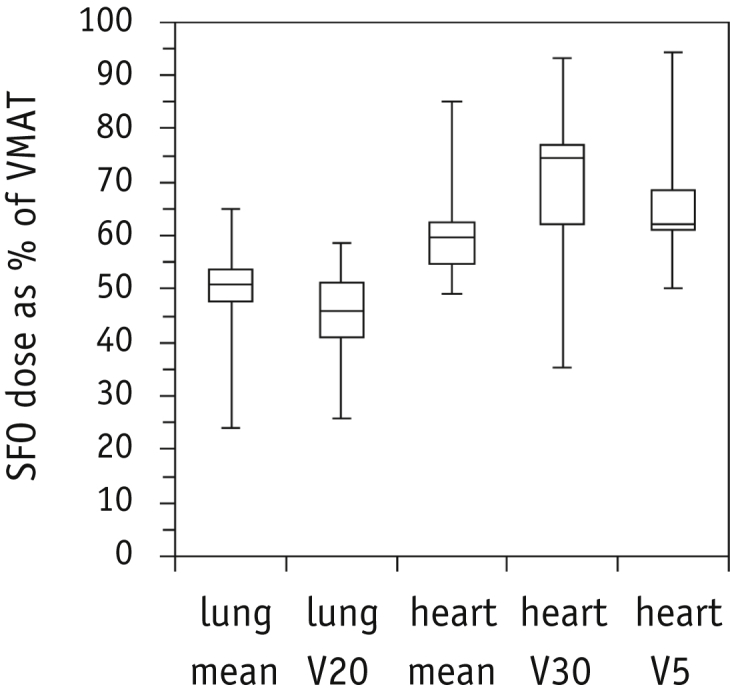
Percentage of dose–volume metric of proton plans compared with photon plans for organs-at-risk. Median and interquartile range are shown in the box plot, with the maximum and minimum values shown as error bars limits. *Abbreviations:* SFO = single-field optimization; VMAT = volumetric arc therapy.

The nominal SFO plans were able to achieve all dose constraints for 20 of the 21 patients (Table E1; available online at www.redjournal.org). The mean heart dose was just exceeded for patient 11 (25.3 Gy), for whom significant overlap of the PTV contour with the heart was present. The target dose coverage was acceptable for all patients, with a D_98%_ of CTV_50Gy_ >95% of the prescribed dose (PD), a median CTV_50Gy_ D_98%_ of 48.9 Gy (range 48.3-49.9), and median GTV D_98%_ of 62.6 Gy (range 62.4-62.9). The proton plans showed a reduced dose to the normal tissue, with a mean median lung value of 6.3 Gy (range 2.5-11.4), lung V_20_ of 6.6% (range 2.5%-17.1%), and maximum cord dose of 23.7 Gy (range 0.1-39.3; Table E1; available online at www.redjournal.org). The higher cord dose of patient 16 allowed greater lung sparing than the VMAT plan. Across all patients, the SFO mean lung dose was only 50.7% (Fig. 3) of dose delivered using photons (median; range 23.9%-64.9%), and this result was statistically significant (*Z* = −4.01; *P*<.001). The SFO mean heart dose was 12.7 Gy (median; range 8.2-25.3), only 59.8% (range 49.1%-84.9%) of the VMAT value (*Z* = 4.01; *P*<.001), and the low dose to the heart contour was also reduced, with a significant reduction in the heart V_5_ for all patients.

### VMAT and SFO plan robustness

**Fig. 4:**
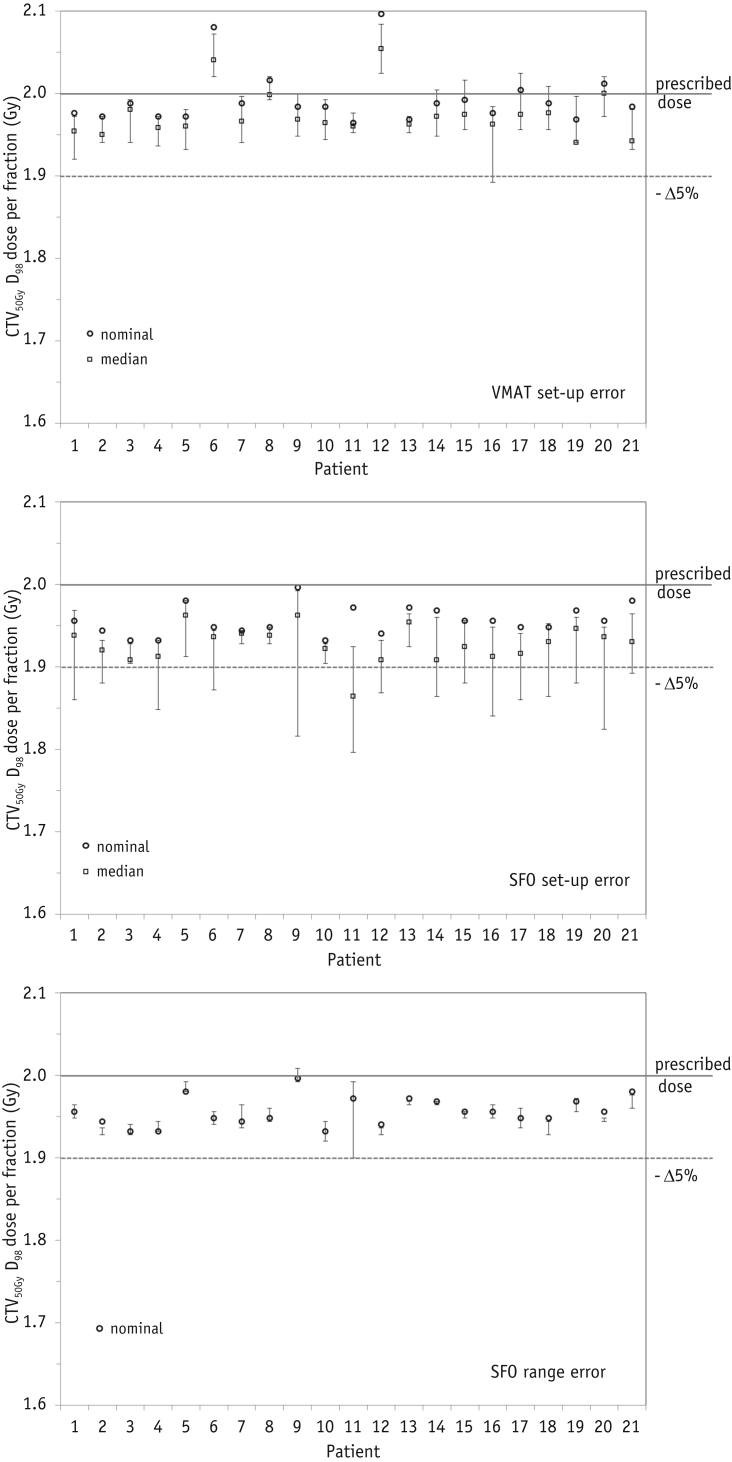
Clinical target volume (CTV) D_98_ dose per fraction for each patient for the nominal plans (circles), with perturbed plans' median dose (squares) and maximum/minimum values (error bars). (Top) Volumetric arc therapy (VMAT) setup error. (Middle) Single-field optimization (SFO) setup error. (Bottom) SFO range error. The prescribed dose and 5% loss in dose per fraction are indicated by horizontal lines.

The CTV_50Gy_ coverage in the presence of setup errors was somewhat reduced (Fig. 4), although for VMAT plans, only 1 patient (patient 16) showed possible dose-per-fraction deterioration of >5% of the PD. This patient had a large overlap (2.5%) of lung tissue in the PTV. For SFO, instances of PD deterioration >5% were observed in 15 of 21 patients. These were most commonly related to displacements 5 mm to the left (9 patients) or 7 mm superiorly (9 patients). Although the absolute minimum SFO dose was always lower (by 0.6%-7.8% of the PD) for each patient than with VMAT. The median perturbed SFO dose was only >5% deterioration level for patient 11, because the proximity of the target and heart generated sharp dose gradients. Proton plan range robustness was acceptable: for all patients, the CTV D_98_ was 95.0% to 100.4% of the PD. The lowest range perturbed dose coverage was again for patient 11.

**Fig. 5:**
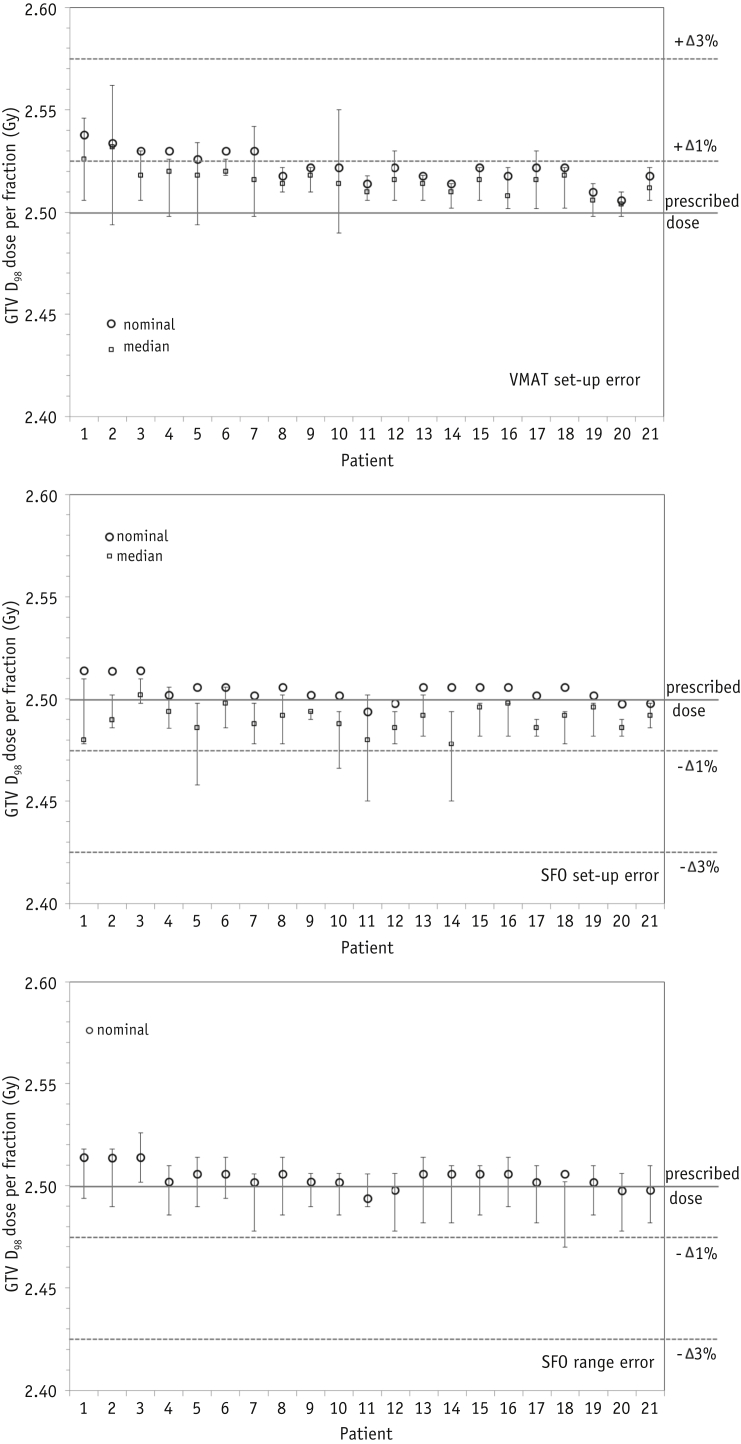
Gross target volume (GTV) D_98_ dose per fraction for each patient for the nominal plans (circles), with perturbed plans' median dose (squares) and maximum/minimum values (error bars). (Top) Volumetric arc therapy (VMAT) setup error. (Middle) Single-field optimization (SFO) setup error. (Bottom) SFO range error. The prescribed dose and 1% and 3% change in dose per fraction are indicated by horizontal lines.

GTV D_98_ plan robustness is illustrated in Figure 5. The VMAT and SFO plans were normalized such that the PTV_62.5Gy_ median dose equaled the PD, although the VMAT plan GTV dose was slightly greater than the SFO dose. Setup error in the VMAT plans gave a minimum GTV D_98_ value of 99.6% to 102.5% the PD, where the median dose reduction from the nominal plan was 0.6% of the PD (range 0.3%-1.6%). The minimum doses were observed for 5-mm displacement right or posteriorly. The setup error in the SFO plans led to a dose of 0.4% to 2% of the PD. The largest dose deteriorations occurred in patients 5 and 14, with 5 mm lateral displacements. Patient 11 (with the sharp dose gradients around the target to reduce the cardiac dose) again had potentially lower minimum GTV D_98_ coverage than that of the other patients. The minimum GTV D_98_ values with the SFO setup errors were lower than the VMAT values by 0.3% to 2.2% of the prescribed GTV dose.

The range errors in the SFO plans resulted in smaller GTV dose deterioration. The values were within 1% of the PD for all but 1 patient, with a median deterioration per fraction of 0.02 Gy (0.8% of the PD).

The reduction in dose to the GTV can be translated to a reduction in the predicted tumor control probability (TCP) using the parameters from Geh et al (2). For the nominal VMAT plans, the TCP was a median of 55.6% (range 54.4%-56.8%); for the SFO technique, the median TCP was 55.1% (range 54.8%-55.3%). When the setup error was included, the TCP for the VMAT plans decreased by 0.3% to 1.5% (median 0.8%), and for perturbed SFO plans, the TCP was decreased by a median of 1.8% (range 1.1%-2.9%), depending on the patient.

For all VMAT plans, even when setup errors were considered, the maximum dose (0.1 cm^3^) to the spinal cord was always less than the required 1.8 Gy/fraction (equivalent to 45 Gy over the entire treatment course), and also always less than the recommended 1.6 Gy/fraction (40 Gy over the treatment course; Figure E1; available online at www.redjournal.org). For the SFO plans, the spinal cord dose was less than the 1.6-Gy dose per fraction limit for all nominal plans and for all but 1 patient for all the perturbed plans. However, patient 16, with a nominal plan spinal cord dose of 1.57 Gy, showed a possible spinal cord dose maximum of 1.8 Gy when the setup error was 7 mm anterior. If this setup error were reproduced at every fraction across the entire treatment course, the resultant dose would be 45.1 Gy.

The doses to lung and heart delivered with setup uncertainties were virtually unchanged for all patients (ie, the same number of patients undergoing VMAT would still not meet the mean heart dose constraint [patients 7, 11, and 17] or lung V_20Gy_ limit [patients 16 and 21]). The lung and heart dose–volume parameters also showed no clinically significant change for the SFO plans with setup error uncertainties.

## Discussion

Our comparison of RT techniques for midesophageal cancer indicated that SFO offers improved cardiopulmonary sparing and equivalent target coverage compared with VMAT. Previous studies have indicated that arc therapy and fixed-field IMRT produce esophageal cancer treatment plans of similar quality 19, 20. The SFO plans significantly reduce the dose to normal tissues, with a median 50% decrease in the mean dose to lung tissue, leading to an expected reduced risk of radiation pneumonitis in these patients. These findings suggest that SFO plans would be favorable for all patients, in particular, for those patients in whom dose escalation with photons is not possible using the current dose constraints. The mean dose to the whole heart was also reduced, with an absolute reduction per patient of 1.9 to 18.7 Gy. Data from a retrospective analysis of breast cancer survivors suggested a 7% reduction in the risk of ischemic heart disease for every 1-Gy decrease in the mean heart dose (21). Also, a significant reduction occurred with SFO in the heart V_5_, which was identified as a dosimetric risk factor in the recent Radiation Therapy Oncology Group 0617 trial (22). However, the volume of heart receiving a greater dose (range V_40Gy_-V_60Gy_) was similar with both techniques, indicating that in cases in which patient anatomy causes close proximity or overlap of organs-at-risk (OARs) with the target volumes, proton therapy will not spare these regions any better than will advanced photon therapy. A recent study of 3-dimensional conformal RT for esophageal cancer suggested that ventricular segments encompassed by the 45-Gy isodose were associated with an increased risk of myocardial ischemia (23). This underlines the need for a correlation of cardiac toxicity with the dose–volume parameters for heart substructures. Knowledge of the safe dose thresholds for cardiac structures will allow for better optimization and comparison of RT plans in the future.

Plan robustness was compared by simulating random setup errors, with a systematic 3.5% range error for the proton plans. Under these conditions, CTV_50Gy_ and GTV D_98_ coverage was acceptable for VMAT and for SFO when a 3.5% range error was taken into account. Random setup error in SFO plans causes a reduction in the dose to the CTV and GTV, for which the magnitude of the dose deterioration is highly dependent on patient anatomy and the direction of the displacement. The errors we simulated were unlikely to be reproduced at every fraction during the treatment course and can be considered as overly pessimistic; some findings have indicated that dose differences are washed out if the treatment course is sufficiently fractionated (24). However, for some patients, strategies to ensure sufficient robustness of the target dose with proton plans would be necessary. This could include optimizing the beam angles for OAR sparing and target robustness for each patient or the use of field-specific margins (25). Large systematic setup errors or range errors >3.5% could reduce the apparent robustness of the SFO planning approach we used, and alternative strategies would be required. Robustness can also be included in the optimization of spot-scanning proton beams (26), although for several of the patients in the present study, the close proximity of OARs and target volumes meant that robust optimization of the dose distribution would likely be challenging. A clinical decision regarding any modification of the target volume margins or OAR sparing would then be required on a patient-by-patient basis.

Patient intrafraction motion owing to respiration could also affect the comparison of the delivered and planned dose distribution for both techniques. Respiration modifies the tumor position and the water-equivalent thickness (WET) of the beam path during the breathing cycle. Because no 4-dimensional (4D)-CT images were available for the patients in the SCOPE database, we measured the WET on a 4D-CT scan of a typical patient with esophageal cancer from our clinic, which showed WET changes of <8 mm along the beam axis crossing the diaphragmatic region. Analysis of the WET variation to select the proton beam angle (using a 4D-CT data set) has been suggested by Chang et al (27) to produce robust SFO plans for thoracic tumors. A planning study using 4D-CT data from lung cancer patients and comparing passive scattering proton therapy (PSPT) and IMRT showed that the dosimetric differences for PSPT were no worse those than for IMRT (28). A similarly rigorous comparison for spot scanning would require detailed knowledge of the time-dependent spot delivery and the interplay with the patient's respiratory cycle. In this context, rescanning methods can conserve target coverage for lung tumors, even for tumor motion of ≤12 mm (29). More significant interfractional dosimetric changes might occur with changes in the baseline respiration amplitude or with changes in gastrointestinal organ filling, which would require careful image-guided verification of the patient anatomy and adaptive replanning during the treatment course.

Inaccuracies in the dose calculation algorithm (in particular, in the presence of heterogeneities) could alter the predicted dose distribution 30, 31, 32. For our study, the maximum heart dose or dose fall-off around the PTV might have been underestimated; however, the GTV coverage and mean lung dose should have been sufficiently accurate. Greater accuracy for dose calculations, in particular for thoracic tumor sites, can be achieved using Monte Carlo and should be introduced for routine treatment planning for both photons and protons.

We compared the physical dose distributions; however, the RBE varies with tissue type and along the path of the proton beam. The net effect might be an underestimation of the RBE (and biological dose), which will be small in the proximal region but can be large in the distal end of the beam, and could significantly underestimate the hot spots in the normal tissues surrounding the tumor (33). For our plan comparison, although the biological lung dose might have been slightly greater than predicted in the proton plans, the advantage in lung sparing was still very clear. In contrast, the dose to heart tissue close to the target could have been underestimated in the proton plans.

## Conclusions

SFO plans achieve significant sparing of lung tissue for all patients with midesophageal cancer compared with VMAT. Further understanding of the mechanism relating the dose to heart toxicity is required, although tumors located adjacent to the heart will always pose a planning challenge, irrespective of the RT modality. The proton plan dose coverage of CTV_50Gy_ and GTV was less robust to random setup errors than were the photon plans, although this was highly patient dependent and can be offset by the potential for OAR sparing. More advanced optimization strategies to ensure adequate target coverage of simultaneous integrated boost proton plans might be required to compensate for large systematic setup errors or range errors >3.5%.

## Figures and Tables

**Table 1:** Dose–volume constraints used for analysis of treatment plans for midesophageal cancer

Dose–volume metric data
PTV_50Gy_
V_95%_ (47.5 Gy) > 95%
PTV_62.5Gy_
V_95%_ (59.4 Gy) > 95%
D_max_ (0.1 cm^3^) <107% (66.9 Gy)
Lung
Mean dose <20 Gy
V_20Gy_ <25%
Heart
Mean dose <25 Gy
V_30Gy_ <45%
CordPRV (5 mm margin)
D_max_ (0.1 cm^3^) <40 Gy (45 Gy permitted)

*Abbreviations:* CordPRV = spinal cord planning risk volume; D_max_ = maximum dose; PTV = planning target volume.

## Supplementary Data

Table E1

Figure E1
