# Supplementary material for: An Analysis of Plan Robustness for Esophageal Tumors: Comparing Volumetric Modulated Arc Therapy Plans and Spot Scanning Proton Planning
Source: Int J Radiat Oncol Biol Phys. 2016 May 1;95(1):199–207. doi: 10.1016/j.ijrobp.2016.01.044 (PMC4838670; doi:10.1016/j.ijrobp.2016.01.044)
Supplement: Table E1 [file mmc1.pdf]

Supplementary Table 1: Dose-volume metrics for nominal plans, patients are listed in order of increasing PTV_{50Gy} size, where unsuccessful dose escalation using VMAT is highlighted.

		VMAT plan								Proton plan							
**Patient No.**	**PTV_{50Gy} volume (cm^3)**	**PTV_{50Gy} D_{95} (%)**	**$PTV_{62.5Gy}$ D_{95} (%)**	**CTV_{50Gy} D_{98} (Gy)**	**GTV D_{98} (Gy)**	**Heart mean (Gy)**	**Cord $0.1cm^3$ (Gy)**	**Lung Mean (Gy)**	**Lung V20 (%)**	**PTV_{50Gy} D_{95} (%)**	**$PTV_{62.5Gy}$ D_{95} (%)**	**CTV_{50Gy} D_{98} (Gy)**	**GTV D_{98} (Gy)**	**Heart mean (Gy)**	**Cord $0.1cm^3$ (Gy)**	**Lung Mean (Gy)**	**Lung V20 (%)**
1	140.1	96.2	97.1	49.4	63.4	19.4	25.4	8.4	5.8	99.8	95.9	48.8	62.8	10.5	1.7	4.3	2.8
2	146.8	97.0	95.5	49.3	63.3	16.0	21.5	10.1	8.0	99.7	98.9	48.5	62.8	10.0	0.0	4.9	4.1
3	195.2	98.2	99.1	49.7	63.2	20.2	31.1	12.1	11.9	98.8	97.8	48.2	62.8	12.8	23.7	6.2	6.3
4	195.6	96.1	96.8	49.3	63.2	19.8	33.3	11.6	10.5	98.2	98.5	48.2	62.5	11.9	28.8	2.9	3.3
5	205.2	96.4	97.7	49.3	63.1	22.2	33.9	10.5	8.5	100.0	99.4	49.5	62.6	13.4	26.6	2.5	2.5
6	218.6	99.8	97.6	52.0	63.2	14.4	32.5	9.6	9.4	99.3	96.8	48.5	62.6	8.6	23.9	5.5	5.0
7	233.4	97.1	95.8	49.7	63.2	**25.6**	34.1	12.3	15.6	99.8	97.5	48.5	62.5	15.6	20.6	6.2	4.0
8	239.0	98.9	96.5	50.4	62.9	16.2	33.7	12.7	14.8	99.7	96.8	48.7	62.6	9.5	21.4	7.2	6.7
9	297.7	96.9	96.9	49.6	63.0	23.9	34.7	13.3	18.1	100.0	98.6	49.9	62.5	14.3	12.3	7.5	8.6
10	301.1	96.8	95.5	49.6	63.0	22.4	27.7	13.2	14.1	99.2	99.4	48.3	62.5	13.4	1.3	6.3	6.0
11	311.7	99.9	99.6	49.1	63.0	**29.8**	33.2	14.0	18.3	99.9	98.3	49.2	62.3	**25.3**	27.8	6.0	9.4
12	329.8	99.7	99.9	52.4	63.0	20.7	34.5	16.7	22.5	97.9	97.5	48.4	62.4	11.3	31.2	8.5	11.1
13	356.1	96.9	95.7	49.2	62.9	15.0	34.2	13.9	15.6	99.1	96.6	49.2	62.6	8.2	24.5	7.0	7.2
14	374.9	98.2	95.9	49.7	62.8	24.0	24.6	13.6	12.9	99.9	95.9	49.1	62.6	13.5	13.3	5.1	5.3
15	405.2	97.6	97.3	49.8	63.0	21.0	34.5	16.2	22.7	99.9	97.2	48.8	62.6	11.4	16.4	8.2	9.4
16	408.8	95.8	95.3	49.4	62.9	19.4	33.2	17.5	**28.8**	97.5	97.6	48.8	62.6	12.7	39.3	11.4	16.8
17	434.2	97.3	95.8	50.1	63.0	**25.5**	27.9	13.7	15.7	98.6	95.8	48.7	62.5	17.4	2.8	6.5	5.8
18	443.1	97.1	96.5	49.7	63.0	21.2	35.4	14.7	17.2	98.9	96.4	48.7	62.6	10.4	23.8	6.3	6.6
19	453.6	96.4	99.7	49.2	62.7	22.0	33.2	14.9	20.2	99.8	99.8	49.1	62.5	12.9	10.3	7.4	8.5
20	544.9	98.8	98.3	50.3	62.6	23.1	36.5	16.0	21.3	99.9	97.9	48.8	62.4	11.9	24.7	8.6	10.0
21	590.8	**92.4**	**92.5**	49.6	62.9	**28.9**	35.4	18.1	**29.7**	99.8	98.4	49.4	62.4	18.5	25.3	11.4	17.1
