# Supplementary material for: An Analysis of Plan Robustness for Esophageal Tumors: Comparing Volumetric Modulated Arc Therapy Plans and Spot Scanning Proton Planning
Source: Int J Radiat Oncol Biol Phys. 2016 May 1;95(1):199–207. doi: 10.1016/j.ijrobp.2016.01.044 (PMC4838670; doi:10.1016/j.ijrobp.2016.01.044)

Supplementary Figure Cord dose (0.1cm^3) robustness for each patient for the nominal plans (circles), with perturbed plans median dose (squares) and maximum /minimum values (error bars). *Top*: VMAT set-up error; *middle*: SFO set-up error and *bottom*: SFO range error.

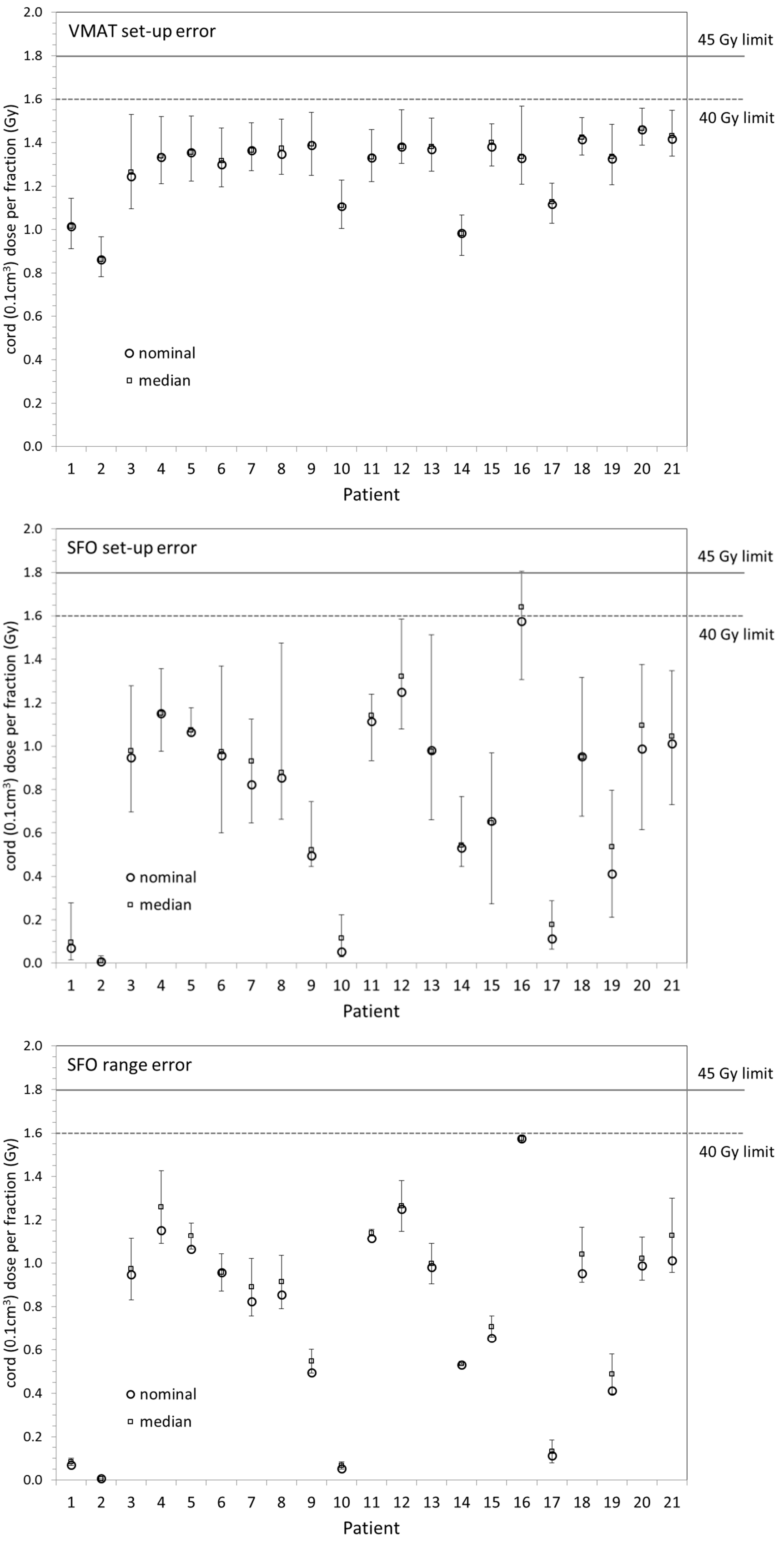

Supplement: Figure E1 [file mmc2.pdf]
